# A Circ-0007022/miR-338-3p/Neuropilin-1 Axis Reduces the Radiosensitivity of Esophageal Squamous Cell Carcinoma by Activating Epithelial-To-Mesenchymal Transition and PI3K/AKT Pathway

**DOI:** 10.3389/fgene.2022.854097

**Published:** 2022-04-29

**Authors:** Junpeng Zhang, Yanyan Yu, Xiaoyang Yin, Lei Feng, Zhe Li, Xiaomeng Liu, Xinshuang Yu, Baosheng Li

**Affiliations:** ^1^ Department of Graduate, Shandong First Medical University and Shandong Academy of Medical Sciences, Jinan, China; ^2^ Department of Radiation Oncology, Shandong Cancer Hospital and Institute, Shandong First Medical University and Shandong Academy of Medical Sciences, Jinan, China; ^3^ Department of Neurology, The First Affiliated Hospital of Shandong First Medical University and Shandong Provincial Qianfoshan Hospital, Jinan, China; ^4^ Department of Oncology, The First Affiliated Hospital of Shandong First Medical University and Shandong Provincial Qianfoshan Hospital, Jinan, China

**Keywords:** circ-0007022, miR-338-3p, neuropilin-1, radiosensitivity, ESCC

## Abstract

Radiotherapy resistance is an important cause of treatment failure in esophageal squamous cell carcinoma (ESCC). Circular RNAs have attracted a lot of attention in cancer research, but their role in ESCC radiosensitivity has not been elucidated yet. Here, we aimed to evaluated the functional impacts of circ-0007022 on ESCC radiosensitivity. In this study, a stable radiotherapy-resistant cell line was established and verified by a series of functional experiments. Subsequently, high-throughput sequencing revealed that circ-0007022 was significantly overexpressed in the radiotherapy-resistant cell line and this conclusion was verified in ESCC patients’ tumor tissues by real-time quantitative PCR. Moreover, loss-of-function and overexpression experiments *in vitro* and *in vivo* revealed that, after irradiation, the abilities of proliferation and migration in circ-0007022-overexpressing stable transgenic strain were significantly higher than that in circ-0007022-knockdown stable transgenic strain. Additionally, RNA Immunoprecipitation, RNA pull-down, luciferase reporter assays, and fluorescence *in situ* hybridization experiments demonstrated the mechanism of how circ-0007022 could sponge miR-338-3p and upregulate downstream target of miR-338-3p, neuropilin-1 (NRP1). Moreover, NRP1 led to poor prognosis for ESCC patients receiving radiotherapy, and NRP1 knock-down enhanced radiosensitivity of ESCC cells. Furthermore, circ-0007022 overexpression activated Epithelial-to-mesenchymal transition and PI3K/Akt pathway, and NRP1 knock-down could reversed this phenomenon. Finally, Akt Inhibitor reversed circ-0007022s role in radiotherapy in ESCC cells. Taken together, the circ-0007022/miR-338-3p/NRP1 axis enhances the radiation resistance of ESCC cells *via* regulating EMT and PI3K/Akt pathway. The new circRNA circ-0007022 is thus expected to be a therapeutic target for ESCC patients.

## Introduction

Esophageal cancer ranks seventh in terms of incidence and sixth overall in mortality, in other words, one of every 18 deaths from tumors is due to esophageal cancer (Sung et al., 2021). Esophageal squamous cell carcinoma (ESCC) is a common pathological type of esophageal cancer, especially in Asia. Surgery, radiotherapy and chemotherapy are the most important treatment methods for esophageal cancer, among which, radiotherapy plays a key role in the local treatment of esophageal cancer (Ajani et al., 2019), but the 5-years survival rate of ESCC is only 21% due to radiotherapy resistance (Murphy et al., 2017). Therefore, finding a molecular target to increase the sensitivity of ESCC radiotherapy is urgently needed.

Circular RNAs (circRNAs) are non-coding RNAs without a 5′-end cap and a 3′-end poly(A) tail, where the 5′ end and 3′ end form a ring structure *via* an end-to-end covalent connection (Yang C. et al., 2021). The expression range of circRNAs is very wide, including in tissues, blood, and exosomes (Fan et al., 2019; Chen et al., 2020; Luo et al., 2021). Moreover, because they are resistant to digestion by nucleic acid exonucleases (RNase R), they are more stable than linear RNAs (Hansen, 2018). In recent years, circRNAs have been demonstrated to play important roles in the occurrence, development, diagnosis, and treatment of tumors, cardiovascular diseases, endocrine diseases, and inflammation, mainly by sponging miRNAs, regulating the translation, directly regulating the transcription, splicing, and expression of parental genes (Li et al., 2015; Du et al., 2017; Liu et al., 2017; Yu et al., 2018; Cao et al., 2020; Zhang et al., 2020). CircRNAs could be reliable and important targets for the treatment of carcinomas. Recent studies had found that circRNAs could modulate the radiosensitivity of various tumors (Li H. et al., 2020; Gao et al., 2021; Yang et al., 2022), however, the interaction between circRNAs and ESCC radiosensitivity has rarely been reported.

In our study, circ-0007022, a new circRNA, was determined to be remarkably upregulated in radiotherapy-resistant ESCC cells and patients, and the phenotypic changes in ESCC cells were closely related to aberrant circ-0007022 expression.

In this study, we aimed to explore the potential functions and mechanisms of circ-0007022, which might be a crucial target to improve existing clinical therapeutic strategies against radiotherapy-resistant ESCC. This study provides new insights into the mechanisms of radiotherapy resistance in ESCC.

## Materials and Methods

### Patients and Tissue Specimens

Two independent cohorts were used in present study as follows: 1) 20 total ESCC patients who received neoadjuvant chemoradiation containing platinum-based chemotherapy drugs were divided into partial response (PR, *n* = 10) and stable disease (SD, *n* = 10) groups based on their imaging examinations before surgery, and tumor tissues of these patients, obtained by endoscopy before they accepted any anti-tumor therapy, were frozen in liquid nitrogen and stored at −80°C; 2) another 22 ESCC patients who had accepted radical radiotherapy with or without concurrent chemotherapy were enrolled in this study, and their baseline characteristics are presented in Supplementary Table S1. Their tumor tissue samples were obtained before they received any treatment, and they were fixed with paraffin for subsequent histological examination. Follow-up was performed over the phone, and the deadline was March 2021. The time between radiotherapy and disease recurrence was defined as progression-free survival (PFS). All patients enrolled in this study accepted radiotherapy treatment at the Shandong Cancer Hospital and Institute (Jinan, China) and had provided written informed consent. The study was approved by the institutional review board of Shandong Cancer Hospital and the Institute for Scientific and Ethical Integrity (approval number: SDTHEC201803).

### Cell Culture and Establishment of Radioresistant Cell Line

The 293T cells and the human ESCC cell lines Kyse30, Kyse150, Kyse410, Kyse450, Kyse510, and TE1 were obtained from the Cell Bank of Shanghai Institutes for Biological Sciences, Chinese Academy of Sciences (Shanghai, China) and authenticated based on short tandem repeats. All cell lines were maintained in Dulbecco’s modified Eagle’s medium (DMEM; Gibco, Grand Island, NY, United States) with 1% penicillin/streptomycin (Biosharp, Hefei, Anhui, China) and 10% fetal bovine serum (Gibco) with 5% CO2 at 37°C. Kyse30 cell were continuously irradiated with a radiation gradient as follows: 2 Gy/f × 3f, 4 Gy/f × 3f, 6 Gy/f × 3f, and 8 Gy/f × 3f. The established radioresistant cell lines were designated as Kyse30R. ESCC cells were incubated with AKT inhibitor MK-2206 (Selleck, Shanghai, China) for 48 h before next experiments, and named as cell-AKT-Inh.

### Colony Formation Assay

Cells (1 × 10^3^) were seeded into 60 mm cell culture dishes (NEST, Wuxi, China), treated with X-rays at doses of 0, 2, 4, or 6 Gy, and then cultured for 14 days to allow colonies to grow. Then, colonies were fixed with 4% paraformaldehyde (Biosharp), stained with 0.1% crystal violet solution (Biosharp), and counted using ImageJ software (National Institutes of Health, Bethesda, MD, United States).

### Cell Survival Assays

Cell survival was analyzed using Cell Counting Kit-8 (CCK-8, Bioss, Beijing, China). Cells with or without irradiation were seeded and incubated in 96-well plates at a density of approximately 3,000 cells/well. At 0, 24, 48, 72, or 96 h, the CCK-8 reagent was added, and the optical absorbance of each sample was measured at 450 nm using an enzyme-labeling instrument (Thermo Fisher Scientific, Waltham, MA, United States) to reflect cell survival.

### Flow Cytometry

At 24 h after a dose of 4Gy irradiation, the cells were collected, washed with cold phosphate-buffered saline (PBS), and fixed overnight at 4°C with pre-cooled 70% ethanol, which was followed by propidium iodide staining. The cells were analyzed using a flow cytometer (BD, Franklin Lakes, NJ, United States). Next, 48 h after a dose of 4Gy irradiation, the cells were collected and analyzed using a Annexin V FITC Apoptosis Detection Kit I (BD), following the manufacturer’s instructions. Subsequently, apoptosis rates was analyzed using FlowJo (v 10.0, BD).

### Western Blot Analysis

Total proteins were extracted using RIPA buffer (KenGEN, Shanghai, China) with a cocktail inhibitor (Beyotime, Shanghai, China). After electrophoresis and transfer, the proteins were blocked with 5% BSA and incubated with primary antibodies as follows: anti-cyclin B1 (Cell Signaling Technology, Danvers, MA, United States; 12231, dilution 1:1,000), anti-cleaved PARP (Cell Signaling Technology; 5625, dilution 1:1,000), anti-cleaved-caspase-3 (Abcam, Cambridge, MA; ab2302, dilution 1:500), anti-γ-H2AX (Abcam; ab26350, dilution 1:1,000), anti-Bax (Cell Signaling Technology; 5023, dilution 1:2000), anti-Argonaute2 (Ago2; Cell Signaling Technology; 2897, dilution 1:1,000), anti-neuropilin-1 (NRP1) (Abcam; ab81321, dilution 1:1,000), anti-E-cadherin (Cell Signaling Technology; 3195, dilution 1:1,000), anti-N-cadherin (Cell Signaling Technology; 5741, dilution 1:500), anti-Akt (Cell Signaling Technology; 4691, dilution 1:1,000), anti-Phospho-Akt (Ser473) (Cell Signaling Technology; 4060, dilution 1:5000), anti-PI3 Kinase catalytic subunit alpha (Abcam; ab40776, dilution 1:1,000), anti-PI3K p85 (phospho Y458)+PI3K p55 (phospho Y199) (Abcam; ab278545, dilution 1:500) and anti-GAPDH (Cell Signaling Technology; 5174, dilution 1:1,000) at 4°C overnight. After incubation with secondary antibodies for 1 h, the membranes were visualized using the ChemiDoc XRS + Gel Imaging System (Bio-Rad, Hercules, CA, United States).

### Immunofluorescence Analysis

For immunofluorescence analysis, the cells were incubated in 24-well plates with or without treatment with radiation at a dose of 6 Gy. Cells were then fixed with 4% paraformaldehyde (Biosharp) for 30min, permeabilized with 1% TritonX-100 (Biosharp) for 30min, blocked with 5% BSA for 1 h, and then incubated overnight at 4°C with primary antibodies (anti-γ-H2AX (Abcam; ab26350, dilution 1:500), anti- Vimentin (Cell Signaling Technology, 5741, dilution 1:500), and primary antibodies were diluted by primary antibody dilution buffer for immunol staining (Beyotime). Subsequently, the cells were incubated for 1 h at 37°C with the secondary antibody. Nuclei were stained with 4′,6-diamidino-2-phenylindole dihydrochloride (DAPI; Solarbio, Beijing, China). Three areas were selected randomly for photography and calculation. Single-staining and merged images were acquired using laser confocal microscopy (LSM 900 with Airyscan 2, Carl Zeiss, Jena, Germany) using Zen 3.0 (blue edition) imaging software (Carl Zeiss).

### Neutral Comet Assay

A neutral comet assay was performed using the CometAssay Kit (Trevigen, Gaithersburg, Maryland, United States). At 4 h after administering radiation therapy at 4 Gy, Kyse30 and Kyse30R cells were mixed with low-density agarose, fixed on the comet slide, and lysed overnight; this was followed by electrophoresis at 21 V for 45 min in a neutral electrophoresis buffer. Gels were then immersed in DNA Precipitation Solution for 30 min at 37°C before staining with SYBR Gold (Invitrogen, Carlsbad, CA, United States). After being photographed using a fluorescence microscope (Carl Zeiss), the olive tail moment was measured using comet score software.

### RNA Extraction, Quantitative Reverse Transcription-Polymerase Chain Reaction, and High-Throughput Sequencing

Total RNA was isolated from cells and tumor tissues using SuperpureTotal RNA Kit (SPARKeasy, Shandong, China), and the reverse transcriptions were performed according to the manuals to obtain the cDNA using miRNA 1st strand cDNA synthesis kit (AG, Changsha, China, for miRNA) and PrimeScript™ RT reagent Kit (Takara, Shiga-Ken, Japan, for mRNA and cirRNA). Then, qRT-PCR was performed in triplicate using SYBR Premix Ex Taq (Takara) and by applying the 2−ΔΔCt method with GAPDH and U6, which were used for normalizing these data. The primers used in the qRT-PCR are listed in Supplementary Table S2. Kyse30 and Kyse30R cells were used to perform High-throughput sequencing to identify circRNAs, according to a previous study (Zhang et al., 2017): Briefly, after RNA quantification and qualification, a total of 3 µg RNA per sample was used as the input material for the RNA sample preparation. Sequencing libraries were generated using the NEBNext^®^ UltraTM RNA Library Prep Kit for Illumina^®^ (NEB, United States). Fragmentation was carried out using divalent cations under elevated temperature in the NEBNext First-Strand Synthesis Reaction Buffer (5X). After first-strand and directional second-strand synthesis, a tailing/adapter ligation approach was performed using the purified cDNA, following amplification. Finally, products were purified, and the Agilent Bioanalyzer 2100 system was used to assess library quality. After cluster generation using the TruSeq PE Cluster Kit v3-cBot-HS (Illumina), library preparations were sequenced using an Illumina Hiseq platform to generate 125 bp/150 bp paired-end reads, followed by Quality control, Mapping to the reference genome, circRNA identification and differential expression analysis for the samples with biological replicates.

### RNase R Digestion, Nucleic Acid Electrophoresis, and Sanger Sequencing

Total RNA (2 µg) was incubated with RNase R (20 U/µL, Lucigen, Wisconsin, United States) for 20 min at 37°C. Control samples were exposed to the same experimental steps as those used for the experimental samples, except for the addition of RNase R. The qRT-PCR products were added to 6 loading buffer (Beyotime) with the nucleic acid dye Gel-Green (Beyotime). After agarose gel (2%) electrophoresis at 60 V for 40 min, DNA fragments were visualized using UV transillumination. Moreover, qRT-PCR amplification products were excised from the agarose gel and purified using a GeneJET Gel Purification Kit (Thermo Fisher Scientific). Subsequently, following the standard approach detailed by Geneseed (Guangzhou, China), the nucleotide sequences of the purified products were determined *via* Sanger sequencing.

### Cell Transfection and Lentiviral Transduction

The circ-0007022-overexpression/knockdown plasmids and the corresponding vectors were successfully constructed using GENE (Shanghai, China), and the miR-338-3p mimic, siRNA-NRP1, and their corresponding negative controls were purchased from GenePharma (Shanghai, China). Kyse150 and TE1 cells (1.5 × 10^5^ cells/well) were seeded into six-well plates and incubated for 8 h before transfection. 20 ul of 1 × 10^7^ TU/ml of plasmids were infected into cells and cultured with 1 ml DMEM with 10% FBS and 6 ug/ml polybrene (GENE) for 48 h, followed by selection using 8 μg/ml puromycin (Thermo Fisher Scientific). 2.5 ul of 20 um miRNA mimics, miRNA labeled with biotin at the 3′ end or siRNAs were injected into cells with 7.5 ul of Lipofectamine 3000 Reagent (Invitrogen) and 500ul of opti-MEM (Gibco), and the transfection efficiency was determined by qRT-PCR analysis after 24 h of transfection. Sequences of mimic, inhibitor, and vectors were listed in Supplementary Table S2.

### Wound Healing Assay

Cells after transfection (2.5 × 10^5^ cells/well) were seeded into 12-well plates and incubated for 24 h. Following 4 Gy irradiation, a 200 µL pipette was used to wound cells, and cells were washed once using PBS to remove cell debris. At 0 and 36 h, cells were photographed, and the wound area was measured.

### Transwell Migration Assays

A transwell assay was also used to assess cell migration. Initially, 2 × 10^4^ cells seeded in the upper chambers of every 8 mm transwell (Millipore, Billerica, MA, United States) were irradiated with 4 Gy of X-rays. The cells in the upper chamber were incubated in serum-free medium, whereas medium with 10% serum was added to the lower chamber. After removing cells on the upper surface of the filter after 48 h, cells attached to the lower chamber were fixed with 4% paraformaldehyde (Biosharp) for 30 min, stained with 0.1% crystal violet solution (Biosharp)for 30 min. Three areas were selected randomly for photography and calculation using an inverted microscope (Carl Zeiss).

### *In vivo* Xenograft Mouse Model

BALB/C nude mice (5–6-week-old) were purchased from Vital River Laboratory (Beijing, China) and subcutaneously injected with transfected cells (1 × 10^7^ transfected cells, *n* = 5 for each group). When tumors reached the intended size, mice were divided randomly into two groups as follows: 1) the irradiation group, which received radiotherapy at a single dose of 8 Gy, and 2) the non-irradiated group. Tumor size was measured using calipers, and the greatest longitudinal diameter (length) and transverse diameter (width) were recorded three times per week. The mice were sacrificed 30 days after injection. Animal experiments were approved by the Institutional Animal Care and Use Committee of Shandong Cancer Hospital and Institute, Shandong First Medical University and Shandong Academy of Medical Sciences (approval number: SDTHEC201803).

### Bioinformatics Analysis

The Circular RNA Interactome (https://circinteractome.nia.nih.gov/) and CircBank Database (www.circbank.cn/) were used to predict circRNAs–miRNA associations. For miRNA–gene interactions, miRDB (http://www.mirdb.org/), miRTarBase (http://mirtarbase.mbc.nctu.edu.tw/php/index.php), TargetScan (http://www.targetscan.org/vert_71/), and miRWalk (http://mirwalk.umm.uni-heidelberg.de/) were used. Gene expression data of human ESCC and corresponding clinical information were obtained from the Cancer Genome Atlas (TCGA) database. All data were downloaded and processed by the R software (version 3.6.1).

### Luciferase Reporter Assay

The wild-type and mutant fragments in the 3′-untranslated region of circ-0007022 and NRP1 related to the miR-338-3p-binding site were generated using GenePharma (Shanghai, China) and subcloned into the pMIR-REPORT vectors. In brief, 5 × 10^3^ cells/well 293T cells transfected with circ-0007022 or circ-0007022-mutant and NRP1 or NRP1-mutant were seeded in 96-well plates and co-transfected with 50 nM miR-NC or miR-338-3p-mimics and the reporter vector using Lipofectamine 2000 (Invitrogen) for 48 h. Then, Firefly and Renilla luciferase activities were measured by a dual-luciferase reporter assay system (Promega, WI, United States) and Relative luciferase activity was normalized to the Renilla luciferase internal control.

### Fluorescence *In Situ* Hybridization

Circ-0007022 and miR-338-3p probes, as well as the FISH kit used in this assay, were all purchased from GenePharma, Sequences of probes were listed in Supplementary Table S2. FISH was performed according to the manufacturer’s instructions. Briefly, 5 × 10^4^ cells/well cells were seeded in 12-well plates and incubated for 24 h. Cells were then fixed with 4% paraformaldehyde (Biosharp) for 15 min, permeabilized with 1% TritonX-100 (Biosharp) for 15 min. Then cells were hybridized at 4°C in hybrid solution containing circ-0007022 and miR-338-3p probes overnight. After washing, cell nuclei were stained with DAPI (Solarbio) at 37°C for 20 min. The single-staining and merged images were captured using a laser confocal microscopy (Carl Zeiss).

### RNA Immunoprecipitation

To analyze the relationships among circ-0007022, miR-338-3p, and NRP1, a RIP experiment was conducted with an RNA Immunoprecipitation Kit (Geneseed, Guangzhou, China). Briefly, 1 × 10^7^ Kyse150 and TE1 cells were lysed in RIP buffer at 4°C for 20 min, and the supernatant incubated overnight with magnetic beads coupled with 5 ug anti-Ago2 (Cell Signaling Technology; 2897) or anti-IgG (Proteintech, Rosemont, United States; 16402-1-AP) at 4°C. Then, after washing, the RNA was extracted from immunoprecipitated complexes and subjected to qRT-PCR.

### RNA Pulldown Assay

Kyse150 and TE1 cells were used to perform the RNA pull-down assay according to a previous study (Phatak and Donahue, 2017). MiR-338-3p-Bio and miR-NC-Bio were constructed by GenePharma (Shanghai, China).

### 5-Ethynyl-2′-Deoxyuridine Assay

The EDU assay was performed using an EDU Kit (Beyotime), according to the manufacturer’s instructions. 5 × 10^4^ cells/well Kyse150 and TE1 cells transfected with si-negative-control or si-NRP1 were seeded onto 12-well plates, irradiated with 4 Gy, and cultured for 48 h, and then, the medium was switched to fresh DMEM supplemented with EDU (50 mmol/L). The cells were then incubated for 2 h, followed by fixation, permeabilization, and EDU staining with Azide 488 (Beyotime). After staining cell nuclei with DAPI, five areas were selected randomly for photography and calculation. And the EDU-positive cells were identified using Zen 3.0 (blue edition) imaging software (Carl Zeiss).

### Immunohistochemistry

NRP1 was detected in 5 µm-thick paraffin sections using the indicated antibodies as previous description (Zeng et al., 2016). Briefly, the sections were incubated with primary antibodies (Abcam; ab81321, dilution 1:500) overnight at 4°C and secondary antibodies (Abcam; ab6789, dilution 1:500) at 37°C for 30 min and then developed using a chromogen (DAB, Biosharp) for 5 min. The staining intensity scoring method was the same as that used in a previous study (Yang Q. et al., 2021).

### Statistical Analysis

Statistical analysis was performed using SPSS (version 22.0; SPSS Inc., Chicago, IL, United States) and GraphPad Prism 8.0.1 (GraphPad Software, San Diego, California, United States). All data were generated from at least three independent experiments, and the results were described as the mean ± standard deviation. Statistical significance was analyzed using a two-tailed Student’s t-test and Mann Whitney U test. Survival rates were calculated using the Kaplan-Meier method, and the Cox proportional hazards regression model was performed for the univariate and multivariate analyses.

## Results

### Confirmation of Radioresistant Cell Line Establishment

**FIGURE 1:**
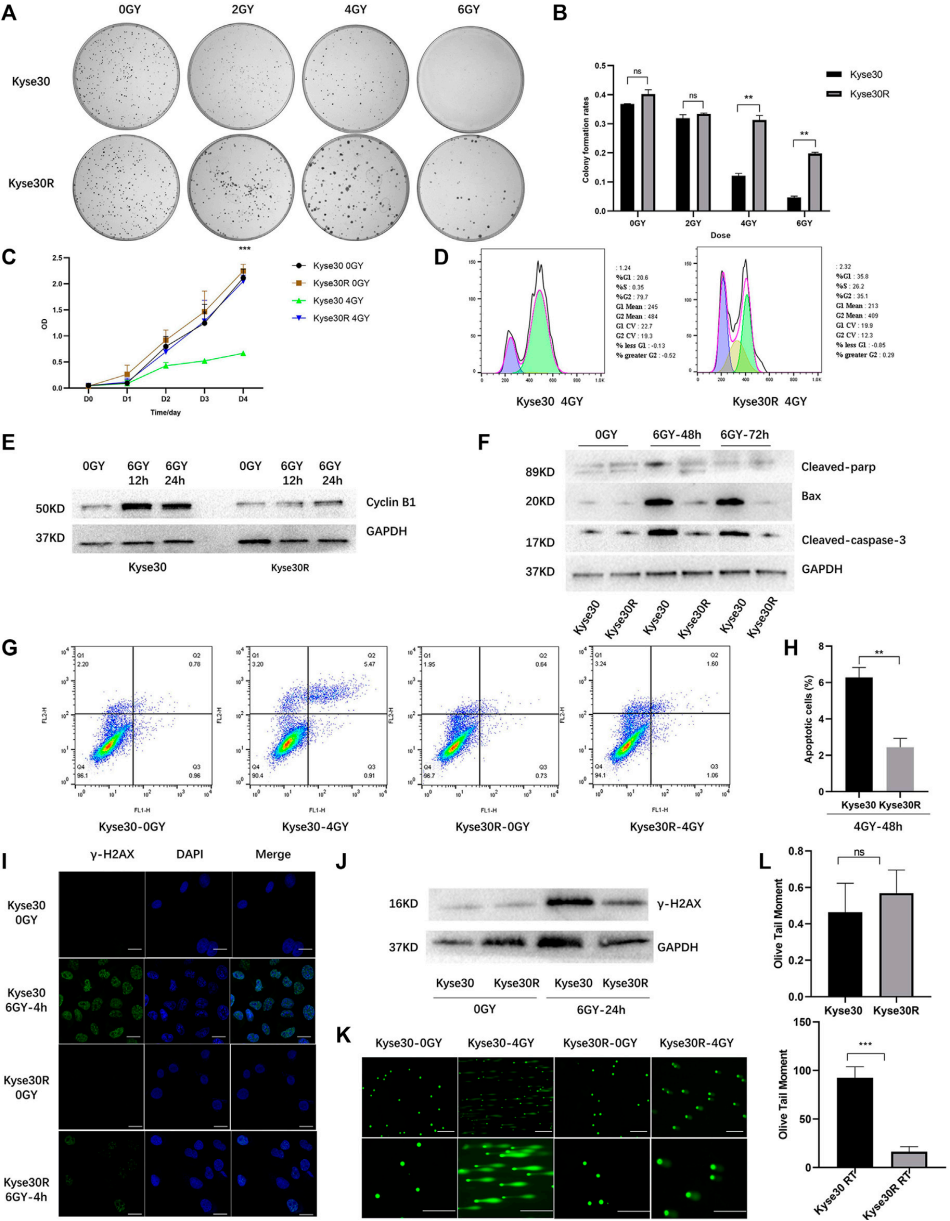
Radioresistant ESCC cell line was established. **(A,B)** Clone formation shown the number of colonies of Kyse30R and Kyse30 cells after irradiation (The Y axis was the ratio of the number of clones to that of cells seeded). **(C)** CCK-8 assays shown the proliferation abilities of Kyse30R and Kyse30 cells after irradiation. **(D,E)** Radiotherapy induced cell cycle arrest in Kyse30R and Kyse30 cells. **(F)** Expressions of several apoptosis-related proteins in Kyse30R and Kyse30 cells. **(G,H)** Radiotherapy induced cell apoptosis in Kyse30R and Kyse30 cells (FL1: Annexin V-FITC, FL2: PI; Q2: Late apoptosis, Q3: Early apoptosis). Immunofluorescence **(I)** (Green, γ-H2AX staining; Blue, DAPI nuclear staining; Scale bar 30 μm), Western blot **(J)** and neutral comet assays **(K, L)** (Scale bar 150 and 100 μm) shown DNA damage in both Kyse30R and Kyse30 cells after radiotherapy. Data are expressed as mean ± SD, ns > 0.05, **p < 0.01, ***p < 0.001.

Kyse30 cells were exposed to radiation based on a gradient, the cumulative dose reached 60 Gy, and the surviving cells, named Kyse30R, were in a stable state. The radiation sensitivity of Kyse30 cells was determined by several functional experiments. Colony formation and CCK-8 assays revealed that after irradiation, the proliferation of Kyse30 cells was significantly reduced, compared to that of Kyse30R cells (*p* < 0.05, Supplementary Table S3, Figures 1A–C). Following X-ray exposure, the proportion of cells in the G2/M phases of the cell cycle was lower in Kyse30R cells than in parental Kyse30 cells (Figure 1D). Simultaneously, expression of the cycle-related molecule cyclin B1 was higher in Kyse30 cells, compared to that in Kyse30R cells (Figure 1E, Supplementary Figure S1A). Further, expressions of several apoptosis-related proteins were significantly higher in Kyse30 cells, compared to that in Kyse30R cells, with radiation (Figure 1F, Supplementary Figure S1B). Moreover, apoptosis analysis revealed that there were fewer Kyse30R apoptotic cells than Kyse30 apoptotic cells after irradiation (*p* < 0.05, Figures 1G,H). In addition, we found that both the frequency of γ-H2AX-positive cells and the expression of γ-H2AX in Kyse30 cells were also significantly increased, compared to those in Kyse30R cells, following exposure to irradiation (*p* < 0.05, Figures 1I,J, Supplementary Figures S1C,D), which indicated more extensive DNA damage in Kyse30 cells. Furthermore, the neutral comet assays revealed DNA double-strand breaks in both Kyse30 and Kyse30R cells after irradiation. Kyse30 cells had longer Olive tails (comets) after electrophoresis, indicating an obviously higher number of double-strand breaks (*p* < 0.001), whereas the damage in Kyse30R cells was not apparent (Figures 1K,L). In summary, the radioresistant cell line was successfully established and could be used for further exploration.

### Identification and Characterization of Circ-0007022 in Radioresistant ESCC Cells

**FIGURE 2:**
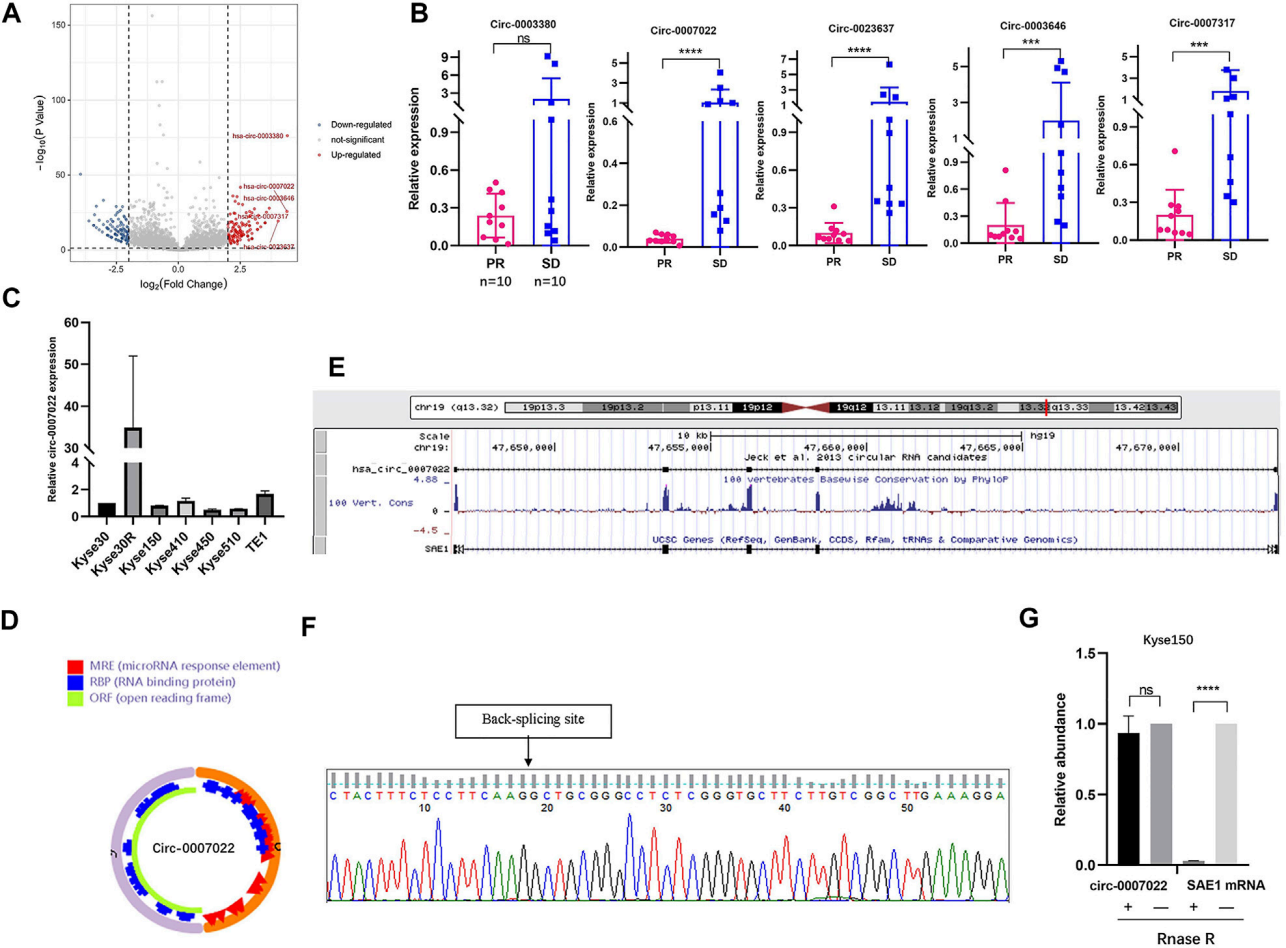
Circ-0007022 expression was upregulated in radioresistant ESCC cell line. **(A)** Volcano plot shown difference in known circRNA expression profiles between Kyse30R and Kyse30 cell lines. **(B)** The qRT-PCR confirmed the different levels of circ-0003380, circ-0007022, circ-0023637, circ-0003646, and circ-0007317 in tumor tissues of ESCC patients. **(C)** The qRT-PCR assay detected the expression of circ-0007022 in multiple ESCC cell lines. **(D,E)** Schematic illustration shown the ring structure of circ-0007022 was composed of two exons (the orange and pink semicircular lines) and the genomic loci circ-0007022. **(F)** Sanger sequencing revealed the “head to tail” splicing sites of circ-0007022 where the arrow pointed. **(G)** RNase R treatment revealed that circ-0007022RNA was resistant to RNase R in ESCC cells, but not mSAE1. Data are expressed as mean ± SD, ns > 0.05, ***p < 0.001, ****p < 0.0001.

After the radioresistant cell line was established, the Kyse30 and Kyse30R cell lines were subjected to high-throughput sequencing to explore the potential circRNAs that might play a role in radiation resistance. There were a total of 1,343 known circRNAs differently expressed in both Kyse30R and Kyse30 cells (log2 fold-change ³1, *p* < 0.05). Among these circRNAs, 668 upregulated and 675 downregulated circRNAs were identified in the Kyse30R cell line, compared to levels in the Kyse30 cell line (*p* < 0.05, Figure 2A). Among the upregulated circRNAs, circ-0003380, circ-0007022, circ-0023637, circ-0003646, and circ-0007317 were ranked as the top five according to the largest fold-change in expression. Then, we measured the level of expression of the top five circRNAs in tumor tissues of patients who received neoadjuvant chemoradiation, and it was found that circ-0007022, circ-0023637, circ-0003646, and circ-0007317 were significantly upregulated in the SD group compared with expression in the PR group (*p* < 0.05, Figure 2B). Thus, combining the fold-change ranking in the high-throughput results and the expression level measured in tumor tissues of patients insensitive to radiotherapy, we finally selected circ-0007022 for further analysis in our study. The expression level of circ-0007022 in the Kyse30R cell line was verified as significantly higher than that in multiple ESCC cell lines (*p* < 0.05, Figure 2C). According to the circRNA annotation in the circBase database (http://www.circbase.org/), back-splicing of circ-0007022 corresponds to 635 bp in length and exon 2 of the SAE1 gene on chromosome 19 (chr19:47646750-47673180) (Figures 2D,E). The back-splicing sequence was identified using Sanger sequencing, as described in the circBase database (Figure 2F). Moreover, the ability to resist RNase R-mediated digestion of circ-0007022, but not of mSAE1, was verified through qRT-PCR (Figure 2G).

### Circ-0007022 Reduces the Radiosensitivity of ESCC Cells *In Vitro* and *In Vivo*

**FIGURE 3:**
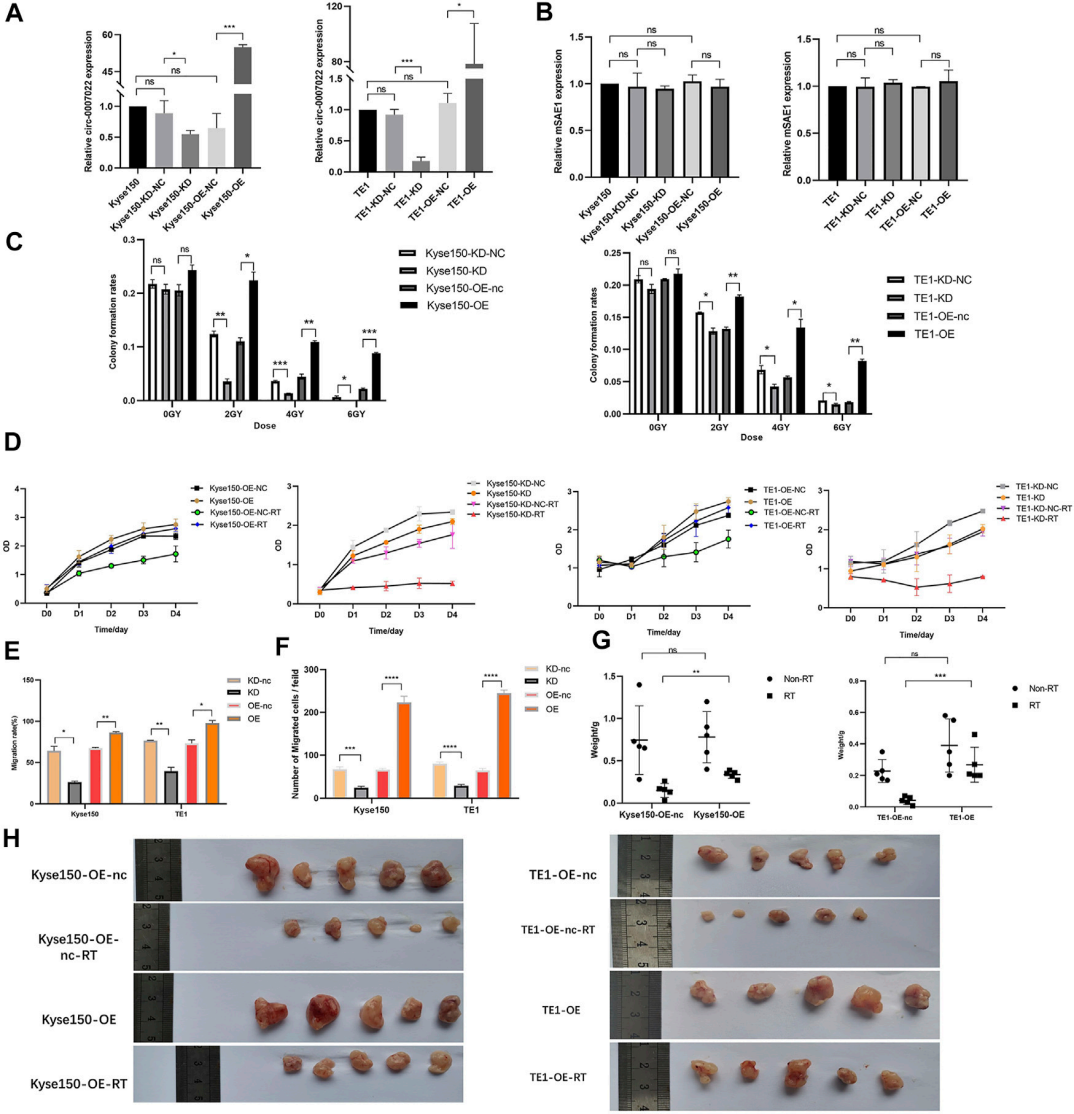
Circ-0007022 reduced radiosensitivity of ESCC cells *in vitro* and *in vivo*. **(A,B)** The qRT-PCR indicated expression levels of circ-0007022 and mSAE1 expression in Kyse150 and TE1 cells after transfection of the overexpression or knockdown plasmids. **(C,D)** Clone formation and CCK-8 assays revealed the proliferation capabilities of Kyse150 and TE1 cells transfected with the overexpression or knockdown plasmids after radiotherapy. **(E,F)** Wound healing and transwell assays assessed cell migration abilities of Kyse150 and TE1 cells, transfected with the overexpression or knockdown plasmids, after radiotherapy. **(G,H)** BALB/C nude mice were treated injected with Kyse150/TE1 cells with transfection of overexpression plasmids or corresponding negative control and received radiation therapy. Tumor wight were detected in each group. Data are expressed as mean ± SD, ns > 0.05, *p < 0.05, **p < 0.01, ***p < 0.001, ****p < 0.0001.

To estimate the function of circ-0007022 in ESCC cells, Kyse150 and TE1 cells were transfected with the circ-0007022-overexpression/knockdown plasmids and the corresponding negative controls, named Kyse150-OE, Kyse150-OE-nc, Kyse150-KD, Kyse150-KD-nc, TE1-OE, TE1-OE-nc, TE1-KD, and TE1-KD-nc. In the aforementioned cells, the expression of circ-0007022 changed significantly with the corresponding transfection (*p* < 0.05), but the expression of mSAE1 showed no obvious change (Figures 3A,B). The proliferation of both cell lines after radiotherapy was enhanced upon circ-0007022 overexpression, whereas circ-0007022 knockdown suppressed their proliferation (Figures 3C,D, Supplementary Figure S2A, Supplementary Table S3). Circ-0007022 overexpression showed the same effect on migration (Figures 3E,F, Supplementary Figure S2B,C). To explore whether circ-0007022 regulates the radiosensitivity of ESCC cells *in vivo*, TE1-OE-nc, TE1-OE, Kyse150-OE-nc, and Kyse150-OE cells were injected into BALB/C nude mice subcutaneously, followed by irradiation and analysis. The overexpression of circ-0007022 remarkably increased the growth and weights of the ESCC tumors with radiation (*p* < 0.01, Figures 3G,H, Supplementary Figure S2D).

### Circ-0007022 Functions as a Sponge for miR-338-3p, and NRP1 is a Direct Target of miR-338-3p

**FIGURE 4:**
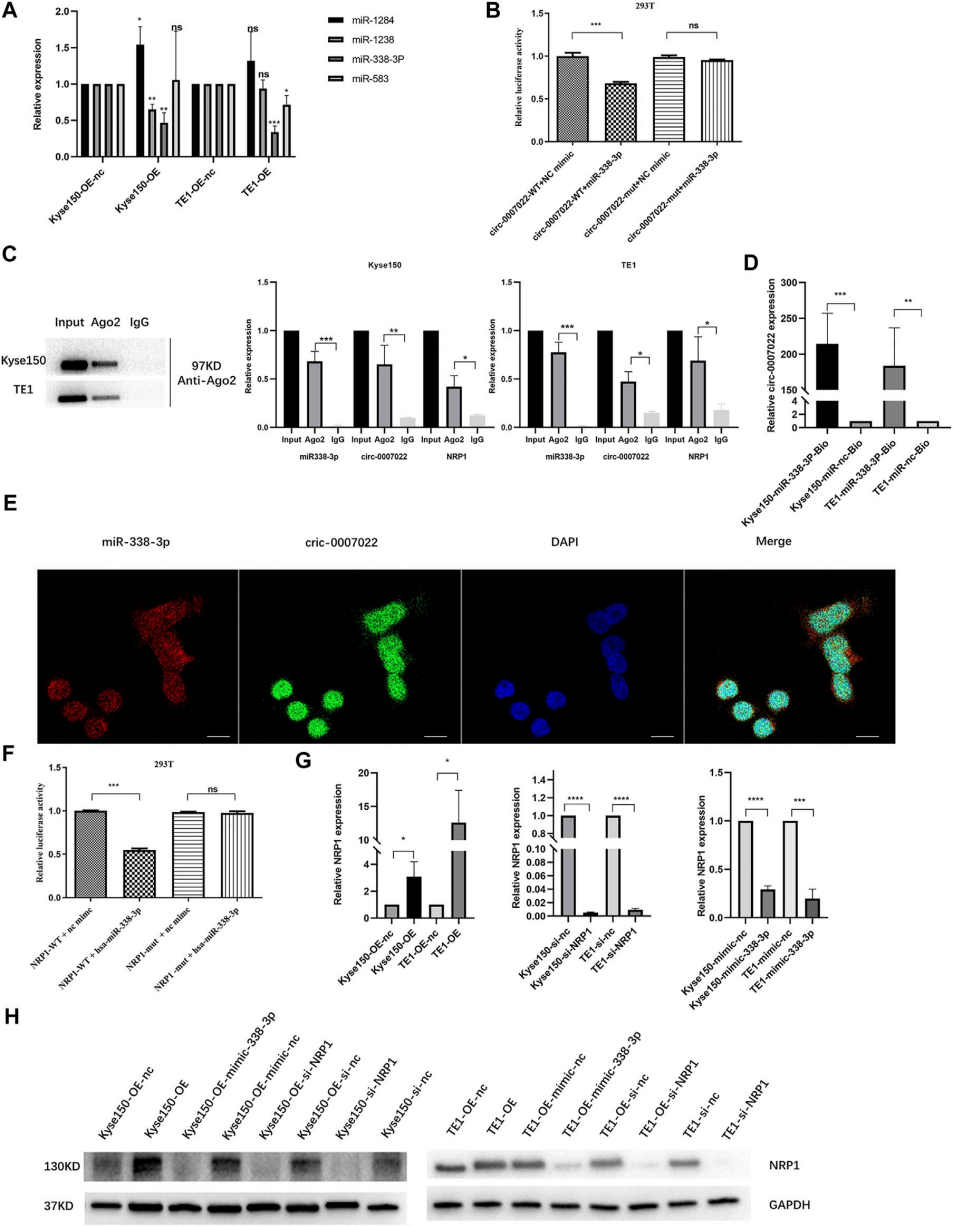
Circ-0007022 functions as a sponge for miR-338-3p, and NRP1 is a direct target of miR-338-3p. **(A)** The qRT-PCR detected relative expression of candidate miRNAs in Kyse150 and TE1 cells transfected with circ-0007022 overexpression or knockdown plasmids. **(B)** The dual luciferase reporter assay indicated that miR-338-3p mimics significantly decreased the luciferase activity of the circ-0007022-WT construct. **(C)** RNA Immunoprecipitation (RIP) analyzed the relationship between circ-0007022 and miR-338-3p, miR-338-3p and NRP1. **(D)** RNA pulldown assays analyzed the relationship between circ-0007022 and miR-338-3p. **(E)** FISH images shown locations of circ-0007022 and miR-338-3p in Kyse150 cells (Red, miR-338-3p probe; Green, circ-0007022 probe; Blue, DAPI nuclear staining; Scale bar 20 μm). **(F)** Luciferase reporter assay confirmed the relationship between miR-338-3p and NRP1. **(G,H)** The qRT-PCR and Western blot revealed NRP1 expression in Kyse150 and TE1 cells after circ-0007022 overexpression, miR-338-3p overexpression or NRP1 knocking down. Data are expressed as mean ± SD, ns > 0.05, *p < 0.05, **p < 0.01, ***p < 0.001, ****p < 0.0001.

RNA interaction prediction tools, including Circular RNA Interactome and Circbank were used to predict the targets of circ-0007022, and the potential interactions between circ-0007022 and four miRNAs are shown (Supplementary Figure S3A). Then, qRT-PCR further revealed that in both Kyse150-OE and TE1-OE cell lines, only the expression of miR-338-3p was negatively altered by circ-0007022, but miR-1283, miR-1248 and miR-583 had no stable relationship with the change of circ-0007022 level in these two cell lines (Figure 4A). To validate our findings, a dual-luciferase reporter assay was performed, which suggested that the miR-338-3p mimic significantly reduced the relative luciferase activity of the wild-type circ-0007022 reporter vector (circ-0007022-WT vector) compared to that with the circ-0007022-MUT vector (*p* < 0.05, Figure 4B, Supplementary Figure S3B). The RIP assay indicated that both circ-0007022 and miR-338-3p were significantly enriched in the immunoprecipitated complexes of Ago2 groups compared to that in IgG groups (Figure 4C), which indicated that circ-0007022 functions as a competing endogenous RNA (ceRNA) by recruiting Ago2 to interact with miR-338-3p. Next, the interaction between circ-0007022 and miR-338-3p was also confirmed by RNA pulldown assay (Figure 4D). Additionally, FISH was performed to validate that circ-0007022 and miR-338-3p were both localized in the same area (Figure 4E). NRP1 was identified as one of the potential downstream targets of miR-338-3p after analysis using four authoritative bioinformatics programs, including miRTarBase, miRwalk, miRDB, and TargetScan (Supplementary Figure S3C). Additionally, the RIP assay and the dual-luciferase reporter assay also validated their interaction (Figures 4C,F, Supplementary Figure S3D). Furthermore, qRT-PCR and western blotting revealed changes in NRP1 levels in Kyse150 and TE1 cell lines following circRNA-0007022 and miR-338-3p overexpression and si-NRP1 transfection (Figure 4G, H). To summarize, these data suggest that circ-0007022 could sponges miR-338-3p to enhance NRP1 translation, and this ceRNA network might be able to explain how circ-0007022 could reduce the radiosensitivity of ESCC.

### Circ-0007022 Activates Epithelial-To-Mesenchymal Transition and PI3K/AKT Pathway by Regulating the miR-338-3p-NRP1 Axis, Leading to Radio-Resistance of ESCC Cells

**FIGURE 5:**
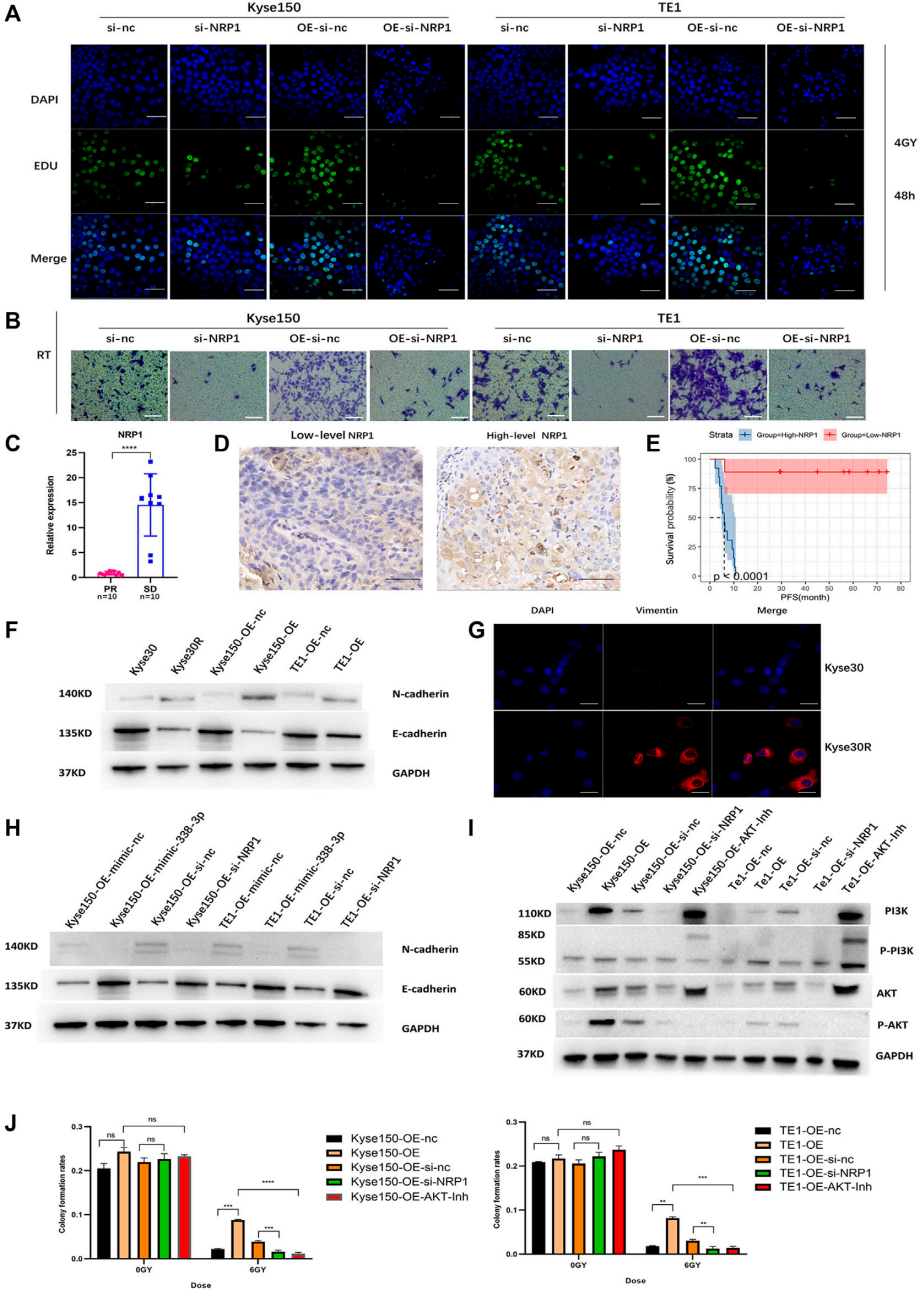
Circ-0007022 activates EMT and PI3K/AKT pathway by regulating the miR-338-3p-NRP1 axis, leading to radio-resistance of ESCC cells. **(A,B)** EDU assay (Green, EDU staining; Blue, DAPI nuclear staining; Scale bar 50 μm) and Transwell assays (Scale bar 50 μm) assessed cell migration and proliferation abilities of Kyse150, TE1, Kyse150-OE and TE1-OE cells, transfected with NRP1 siRNAs, after radiotherapy. **(C)** The qRT-PCR detected the expression level of NRP1 in tumor tissues of patients. **(D)** Immunohistochemical staining image of NRP1 was acquired on tumor tissues from High/Low level group (Scale bar 50 μm). **(E)** Kaplan-Meier analysis showed that the level of NRP1 was predictive of PFS in ESCC patients receiving radiotherapy (PFS, progression-free survival). **(F,G)** Western blot and Immunofluorescence (Red, Vimentin staining; Blue, DAPI nuclear staining; Scale bar 30 μm) analyzed expression change of EMT-related proteins (N-cadherin, E-cadherin and Vimentin) in ESCC cells with circ-0007022 overexpression. **(H)** The relative expressions of N-cadherin and E-cadherin in Kyse150 or TE1 cells after indicated transfection were analyzed by western blot. **(I)** Western blot analyzed expression change of PI3K/AKT pathway related proteins (PI3K, p-PI3K, AKT and p-AKT) in ESCC cells after indicated transfection or AKT inhibitor incubation. **(J)** Clone formation shown the proliferation ability of Kyse150-OE and TE1-OE cells, with si-NRP1 transfection or AKT inhibitor incubation, after irradiation. Data are expressed as mean ± SD, ns > 0.05, **p < 0.01, ***p < 0.001, ****p < 0.0001.

**FIGURE 6:**
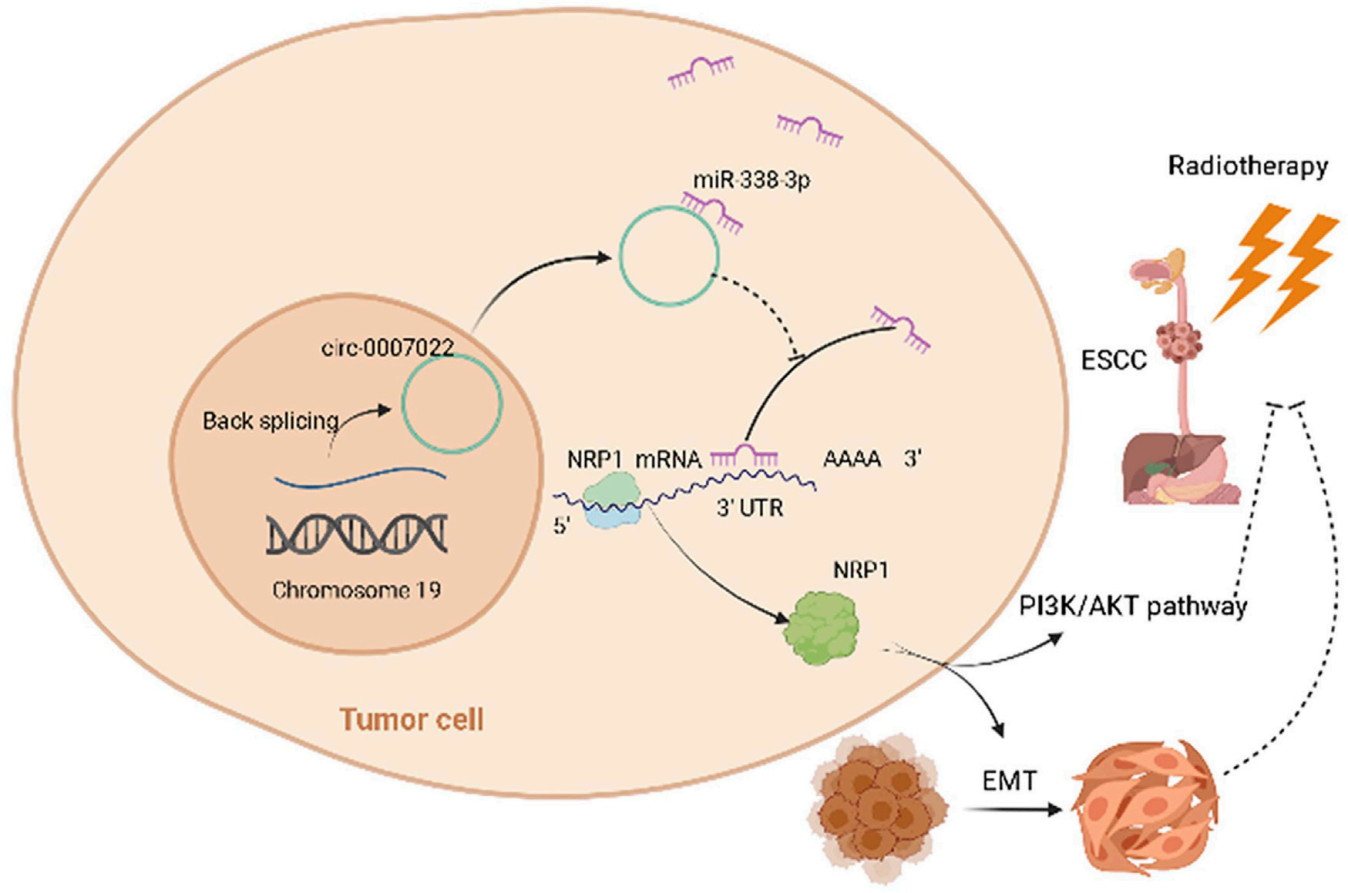
Schematic of the mechanisms of circ-0007022 in the regulation of radiotherapy resistance in ESCC. Circ-0007022/miR-338-3p/NRP1 axis promotes ESCC radiotherapy-resistance by activating EMT and PI3K/AKT pathway.

The EDU and transwell migration assays indicated that silencing NRP1 remarkably decreased the proliferation and migration of Kyse150, TE1, Kyse150-OE and TE1-OE cells exposed to radiation (*p* < 0.05, Figures 5A,B, Supplementary Figure S4A,B). Subsequently, we analyzed the relationship between the NRP1 level and the prognosis of ESCC patients receiving radiotherapy. On one hand, for ESCC patients administered neoadjuvant chemoradiation, NRP1 was significantly upregulated in the SD group compared with levels in the PR group (*p* < 0.05, Figure 5C). On the other hand, for ESCC patients receiving radical radiotherapy with or without concurrent chemotherapy, those with high NRP1 expression had a more unfavorable PFS than those with lower NRP1 expression (*p* < 0.0001, Figures 5D,E). Multivariate analysis showed that NRP1 and AJCC staging were independent prognostic indicators for PFS (Supplementary Table S1). In order to deeply explore the relationship between NRP1 and radiosensitivity in ESCC, GO (Gene Ontology) analysis (Supplementary Figure S4C) and gene set enrichment analysis (Supplementary Figure S4D) were performed and found that high NRP1 expression was closely and positively related to EMT activation, indicating EMT might account for the role of NRP1 in ESCC radiotherapy resistance. As expected, western blotting demonstrated increased protein levels of a mesenchymal molecular marker (N-cadherin) but decreased protein levels of an epithelial molecular marker (E-cadherin) following circ-0007022 overexpression (Figure 5F, Supplementary Figure S4E). Moreover, another mesenchymal molecular marker (Vimentin) was also be found overexpressed in the Kyse30R cell line (Figure 5G). Additionally, rescue experiments revealed that the co-transfection of miR-338-3p mimic or si-NRP1 significantly reversed the positive effect on EMT induced by circ-0007022 upregulation (Figure 5H, Supplementary Figure S4F). Furthermore, inhibition of the PI3K/Akt/mTOR signaling pathway had been found to enhance radiosensitivity of tumor cells. Thus, western blot (Figure 5I, Supplementary Figure S4G) was performed and shown that, p-AKT was up-regulated with circ-0007022 overexpression, but down-regulated with silencing NRP1 or AKT inhibitor incubated. PI3K had the same changes as p-AKT with circ-0007022 or NRP1 changed. Otherwise, in TE1 cells, AKT inhibitor would enhance the expression of PI3K and p-PI3K, and si-NRP1 also could upregulate the level of p-PI3K. However, there was little difference of the expression of AKT between groups in both two cells or the expression of p-PI3k between groups in Kyse150 cells. Additionally, rescue experiments indicated that both si-NRP1 and AKT inhibitor could reversed radioresistant ability of Kyse150-OE and TE1-OE cells (Figure 5J, Supplementary Figure S4H). Collectively, the circ-0007022/miR-338-3p/NRP1 axis promotes ESCC radiotherapy-resistance by activating EMT and PI3K/AKT pathway (Figure 6).

## Discussion

As important non-coding RNAs, circRNAs were considered to have no regulatory effects in the early days of their discovery (Hsu and Coca-Prados, 1979). With the development of RNA sequencing technology, many circRNAs have been identified and been found that they can take part in biological processes, as with linear RNAs (Goodall and Wickramasinghe, 2021). ESCC studies have shown that multiple circRNAs are related to metastasis, migration, invasion, progression, and diagnosis (Hu et al., 2019; Ma et al., 2020; Shi et al., 2020; Zhou et al., 2021). Nevertheless, there is very little research on the role of circRNAs in ESCC radiosensitivity, and there are no reports on the role of circ-0007022 in cancer. Hence, in this study, we first time explored the promoting effect of circ-0007022 on radiotherapy resistance and verified the regulatory network of circ-0007022/miR-338-3p/NRP1 in ESCC.

In this study, we found that patients who were not sensitive to neoadjuvant chemoradiation had a high level of circ-0007022, a new circRNA, indicating that circ-0007022 might be involved in the radioresistance of ESCC. In contrast with a similar previous study (Liu J. et al., 2019), we chose two different ESCC cell lines to explore the function of circ-0007022. Functionally, the downregulation of circ-0007022 inhibited the migration and proliferation of cells after radiation *in vitro* and *in vivo*, whereas circ-0007022 overexpression increased resistance to radiation in ESCC cells. Consequently, our results indicated that circ-0007022 might play a key role in ESCC radiosensitivity. In recent years, studies have found that the most common form of circRNA acts as an miRNA sponge to establish a circRNA/miRNA/mRNA axis (Ng et al., 2018). For example, by sponging miR-200c-3p, circ-001783 regulates the progression of breast cancer (Liu Z. et al., 2019). CircNRIP1 promotes cervical cancer migration and invasion by sponging miR-629-3p and regulating the PTP4A1/ERK1/2 pathway (Li X. et al., 2020). Thus, dual-luciferase reporter assays, RIP, pull-down, and FISH assays were used in the present study to prove the direct association between circ-0007022 and miR-338-3p. We also found that miR-338-3p was significantly downregulated in ESCC cells overexpressing circ-0007022. Moreover, the expression of mSAE1 in ESCC cells overexpressing circ-0007022 was similar to that in ESCC cells after transfection of the corresponding negative control, and thus we reasoned that radiosensitivity was affected by circ-0007022 sponging miR-338-3p, instead of mSAE1 sponging miR-338-3p. In multiple cancers, miR-338-3p functions as a tumor suppressor (Sun et al., 2018; Wang and Qin, 2018; Song et al., 2021). Moreover, miR-338-3p is sponged by circRNAs in tumors, which is consistent with our findings (Xiong et al., 2019; Liu et al., 2020a; Liu et al., 2020b). These findings show that circ-0007022 might exert a negative effect on ESCC radiosensitivity by sponging miR-338-3p.

The ceRNA hypothesis represents a new model of gene expression regulation. Compared with miRNA, the ceRNA regulation network is more sophisticated and complex, and circRNAs can act as ceRNAs to regulate miRNA target gene expression (Salmena et al., 2011). NRP1, a non-tyrosine kinase transmembrane receptor, is highly expressed in many cancers, especially in gastrointestinal cancers, promoting metastasis and proliferation (Li et al., 2016; Zhang et al., 2018; Ding et al., 2019; De Vlaeminck et al., 2020; Abdullah et al., 2021; Wang et al., 2021). A series of experiments indicated that NRP1 is a functional target of miR-338-3p. Meanwhile, circ-0007022 overexpression also increased the expression of NRP1 at the mRNA and protein levels, whereas miR-338-3p mimics had an opposing effect, indicating that the ceRNA network circ-0007022-miR338-3p-NRP1 was stable. Silencing NRP1 led to a decrease in the proliferation and migration of ESCC cells exposed to radiation, and patients with higher NRP1 expression in ESCC tissues exhibited shorter PFS after radiotherapy. All these confirmed our conjecture, specifically that the mechanism of radiation resistance might be associated with an increase in NRP1 expression. In addition, conforming with our bioinformatics analysis, previous research found that NRP1 might modulate EMT to not only contribute to the growth and metastasis of tumors (Chen et al., 2018; Ma et al., 2019; Ding et al., 2020), but also induce drug-resistance and radiation resistance in cancers (Abdullah et al., 2021; Cong et al., 2021). Our results showed that the expression levels of EMT-related markers, including N-cadherin and vimentin, were significantly increased, accompanied by a decrease in E-cadherin, in cells with radiation resistance and circ-0007022 overexpression. Subsequently, silencing NRP1 reversed the positive effect of circ-0007022 upregulation on EMT. Furthermore, it is known that NRP1 was associated with AKT pathways (Jin et al., 2020), we found circ-0007022-OE upregulated the expression of p-AKT and PI3K, and si-NRP1 may reverse this phenomenon, suggesting that NRP1 upregulated by circ-0007022 could activate PI3K/AKT pathway. Moreover, AKT inhibitor decreased radiation resistance of circ-0007022 overexpression ESCC cells, indicating circ-0007022 could regulate radiosensitivity through PI3K/AKT pathway. In addition, in TE1 cells, the phenomenon that AKT inhibitor and si-NRP1could enhance the expression of p-PI3K might be the result of negative feedback after AKT pathways inhibited. All of these results indicate that the ceRNA network, composed of circ-0007022, miR-338-3p, and NRP1, could affect sensitivity of radiotherapy in ESCC by activating EMT and PI3K/AKT Pathway. In addition, previous studies have found that NRP1 could affect the sensitivity of radiotherapy through NF-κB pathway in non-small cell lung cancer (Dong et al., 2015), so whether other pathways also involved in the process of circ-0007022/miR-338-3p/NRP1 axis’ regulation of radiotherapy resistance in ESCC still needs further research explore in the further.

To sum up, our results first time corroborate the hypothesis that the circ-0007022/miR-338-3p/NRP1 axis leads to radiation resistance by activating EMT and PI3K/AKT pathway in ESCC, establishing a circRNA-miRNA-mRNA network. These findings provide new evidence that circRNAs exert functions in ESCC and present several new potential therapeutic targets for reducing radiotherapy failure in patients with ESCC.

## Data Availability

The datasets presented in this study can be found in online repositories. The names of the repository/repositories and accession number(s) can be found below:https://www.ncbi.nlm.nih.gov/bioproject/PRJNA808226.
