# Supplementary figures and images for: A Circ-0007022/miR-338-3p/Neuropilin-1 Axis Reduces the Radiosensitivity of Esophageal Squamous Cell Carcinoma by Activating Epithelial-To-Mesenchymal Transition and PI3K/AKT Pathway
Source: Front Genet. 2022 Apr 29;13:854097. doi: 10.3389/fgene.2022.854097 (PMC9100939; doi:10.3389/fgene.2022.854097)

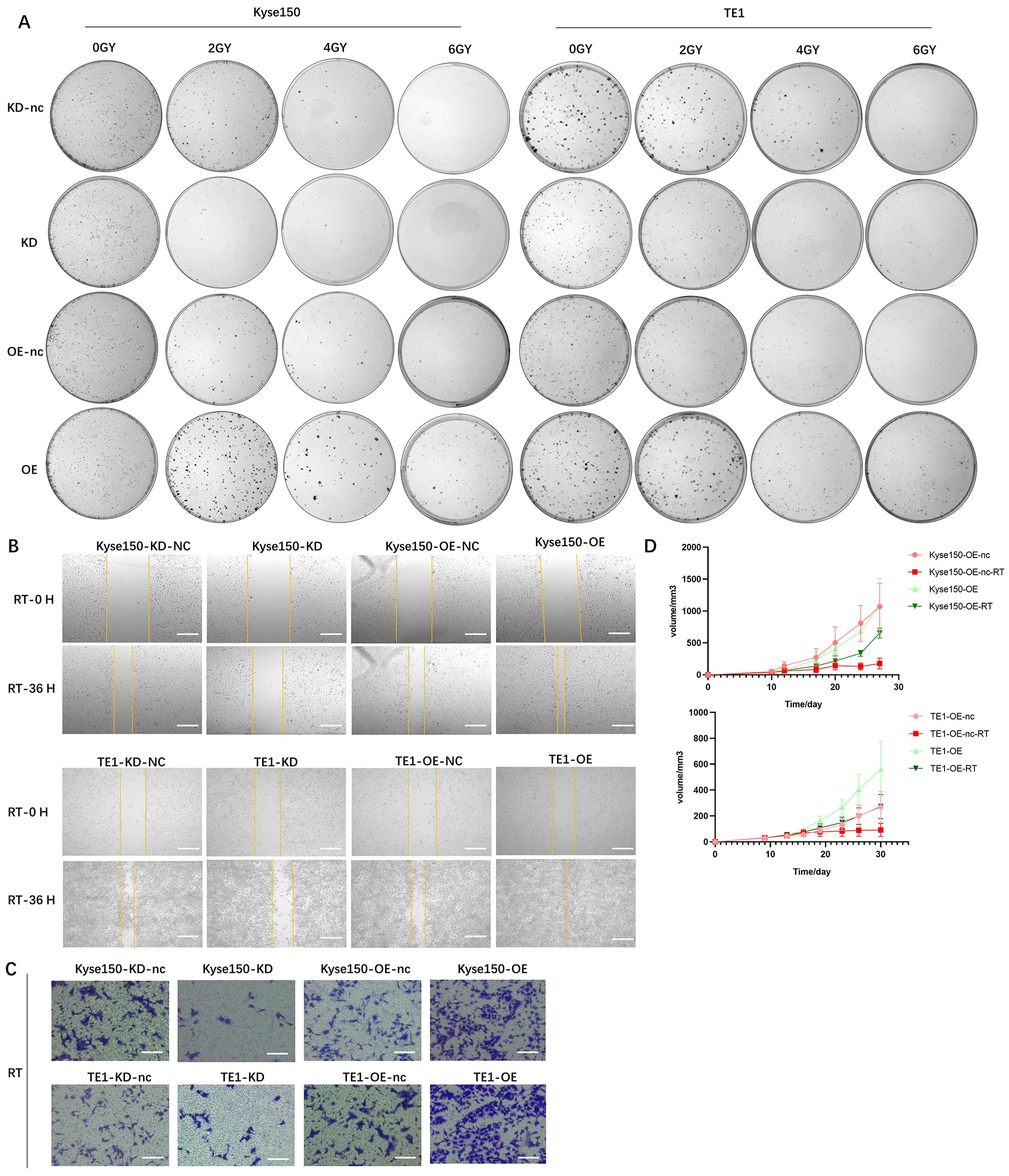

Supplement: Supplementary file 2 [file Image2.jpg]

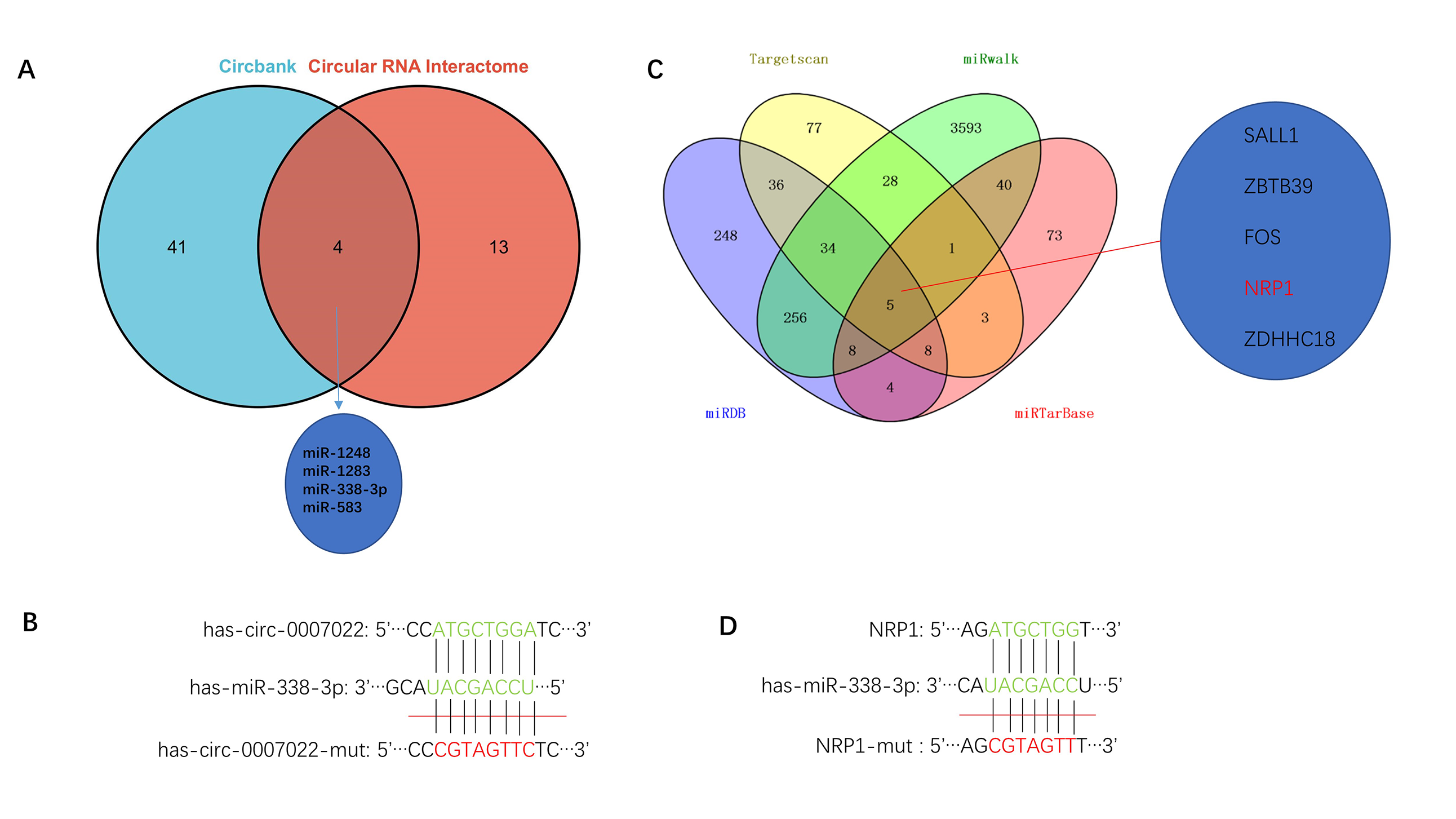

Supplement: Supplementary file 4 [file Image3.TIF]

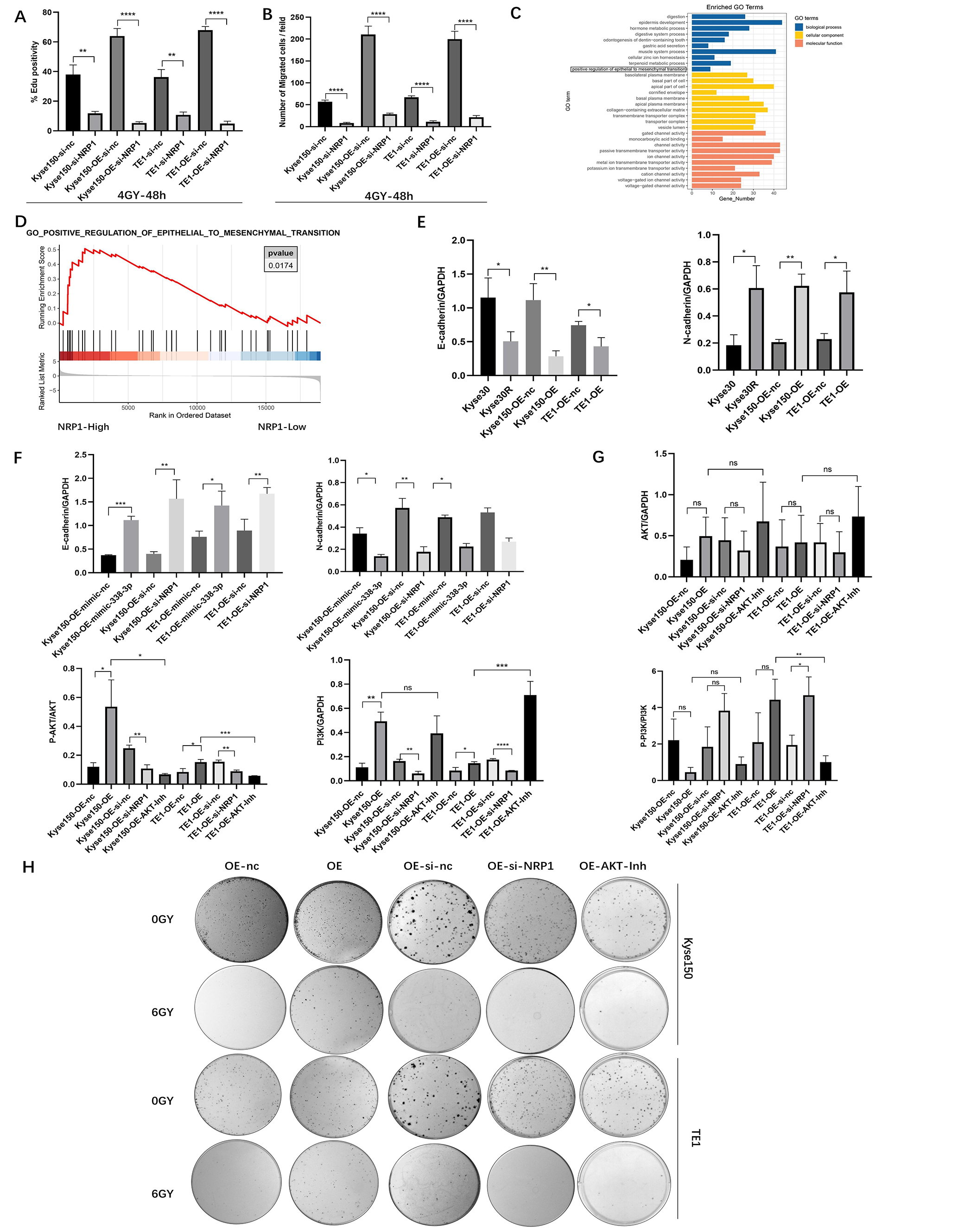

Supplement: Supplementary file 5 [file Image4.TIF]

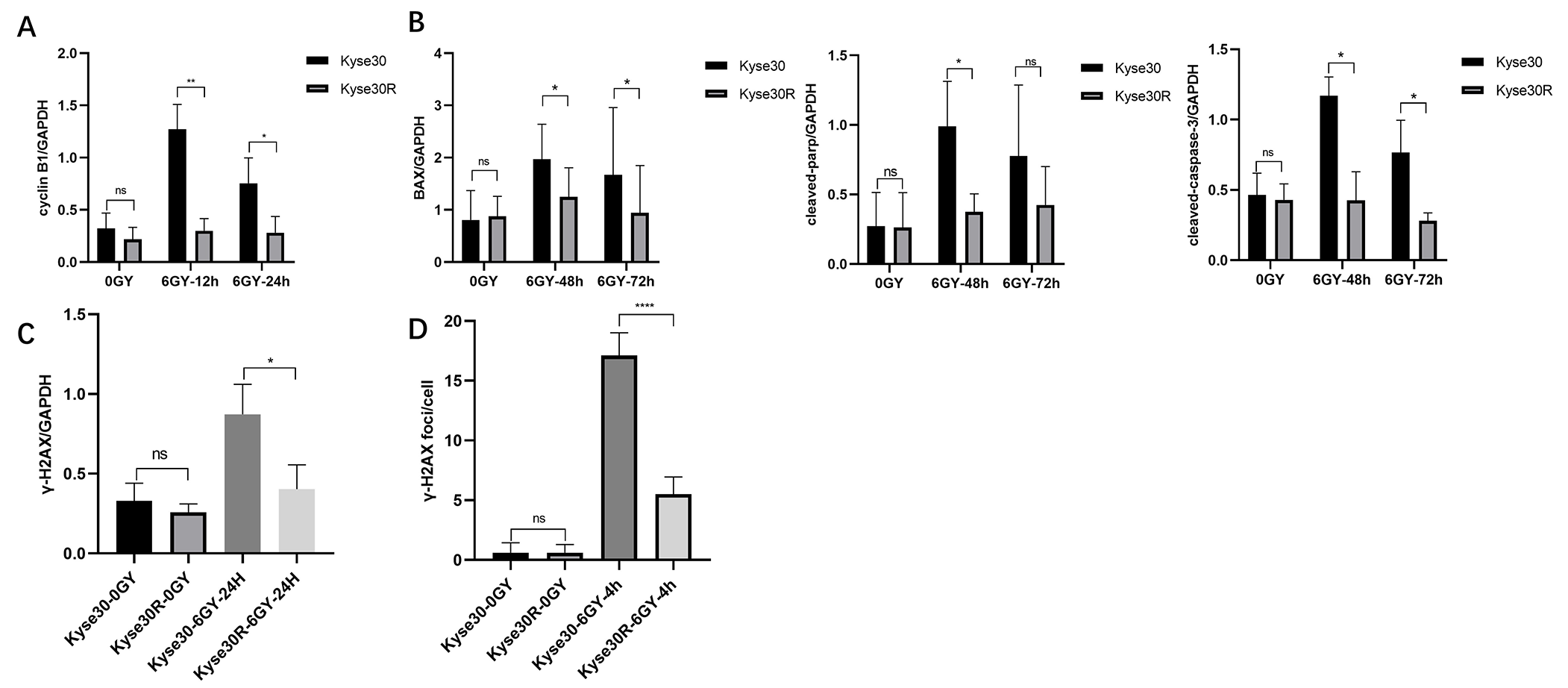

Supplement: Supplementary file 7 [file Image1.tif]
