# Supplementary material for: A Circ-0007022/miR-338-3p/Neuropilin-1 Axis Reduces the Radiosensitivity of Esophageal Squamous Cell Carcinoma by Activating Epithelial-To-Mesenchymal Transition and PI3K/AKT Pathway
Source: Front Genet. 2022 Apr 29;13:854097. doi: 10.3389/fgene.2022.854097 (PMC9100939; doi:10.3389/fgene.2022.854097)
Supplement: Supplementary file 8 [file Table4.DOCX]

**Figure S1** Confirmation of radioresistant cell line establishment. **(A)** The expression of cyclin B1 in Kyse30 and Kyse30R cells with or without radiotherapy. **(B)** Expressions of several apoptosis-related proteins in Kyse30R and Kyse30 cells with or without radiotherapy. **(C, D)** Expressions of γ-H2AX in Kyse30R and Kyse30 cells with or without radiotherapy. Data are expressed as mean ± SD, ns>0.05, *P < 0.05,**P < 0.01,****P < 0.0001.

**Figure S2** Circ-0007022 reduced radiosensitivity of ESCC cells *in vitro* and *in vivo*.**(A)** Clone formation revealed the proliferation capabilities of Kyse150 and TE1 cells transfected with the overexpression or knockdown plasmids after radiotherapy. **(B)** Wound healing (Scale bar 300μm) assessed cell migration abilities of Kyse150 and TE1 cells, transfected with the overexpression or knockdown plasmids, after radiotherapy. **(C)** Transwell assays (Scale bar 50μm) assessed cell migration abilities of Kyse150 and TE1 cells, transfected with the overexpression or knockdown plasmids, after radiotherapy. **(D)** Tumor volume were detected in each group.

**Figure S3** Interaction between Circ-0007022 and miR-338-3p, miR-338-3p and NRP1. **(A)** Schematic illustration shown the intersection of miRNAs targeting circ-0007022 predicted by circbank and Circular RNA Interactome. **(B)** Schematic representation of potential binding sites of miR-338-3p on circ-0007022 3’-UTR and mutant binding sites. **(C)** Schematic diagram exhibited the overlap of miR-338-3p target predicted by miRTarBase, miRwalk, miRDB, and TargetScan. **(D)** Schematic shown that NRP1 3’UTR contain complementary sequence of miR-338-3p.

**Figure S4** The circ-0007022/miR-338-3p/NRP1 axis activates EMT and PI3K/AKT pathway. **(A, B)** EDU assay and Transwell assays assessed cell migration and proliferation abilities of Kyse150, TE1, Kyse150-OE and TE1-OE cells, transfected with NRP1 siRNAs, after radiotherapy. **(C)** GO analysis of NRP1 related pathways in TCGA ESCC dataset. **(D)** Gene Set Enrichment Analysis of the relationship between the expression level of NRP1 and EMT-related gene signatures in the TCGA ESCC dataset. **(E)** Western blot analyzed expression change of N-cadherin and E-cadherin in ESCC cells with circ-0007022 overexpression. **(F)** Western blot analyzed expression change of N-cadherin and E-cadherin in Kyse150-OE and TE1-OE cells, transfected with mimic-338-3p or si-NRP1. **(G)** Western blot analyzed expression change of PI3K/AKT pathway related proteins (PI3K, p-PI3K, AKT and p-AKT) in Kyse150 and TE1 cells after indicated transfection or AKT inhibitor incubation. **(H)** Clone formation shown the proliferation ability of Kyse150 and TE1 cells with indicated transfection or AKT inhibitor incubation, after irradiation. Data are expressed as mean ± SD, ns>0.05, *P < 0.05,**P < 0.01,***P < 0.001,****P < 0.0001.
